# Surveillance of Twitter Data on COVID-19 Symptoms During the Omicron Variant Period: A Sentiment Analysis

**DOI:** 10.2196/66237

**Published:** 2025-09-23

**Authors:** Kaiyue Zhang, Zhaojin Guo, Yujie Ai, An-Ran Li, Anlin Li, Ziyu Liu, Yittie Yi Ting Tse, Xinyu Zhou, Taoran Liu, Chuxi Xiong, Jian Huang, Wai-kit Ming

**Affiliations:** 1 School of Public Health, Li Ka Shing Faculty of Medicine, University of Hong Kong, Hong Kong, China (Hong Kong); 2 Department of Paediatrics and Adolescent Medicine, School of Clinical Medicine, University of Hong Kong, Hong Kong, China (Hong Kong); 3 Department of Infectious Diseases and Public Health, Jockey Club College of Veterinary Medicine and Life Sciences, City University of Hong Kong, To Yuen Building, 31 To Yuen Street, Hong Kong, China (Hong Kong), 852 34426956; 4 Department of Family Medicine and Primary Care, Li Ka Shing Faculty of Medicine, School of Clinical Medicine, University of Hong Kong, Hong Kong, China (Hong Kong); 5 Department of Pharmacology and Pharmacy, Li Ka Shing Faculty of Medicine, University of Hong Kong, Hong Kong, China (Hong Kong); 6 Department of Medical Record, Peking University Third Hospital, Beijing, China; 7 Department of Health, The Government of the Hong Kong Special Administrative Region, Hong Kong, China (Hong Kong); 8 Department of Science, University of Waterloo, Waterloo, ON, Canada; 9 Institute for Human Development and Potential (IHDP), ASTAR, Singapore, Singapore; 10 Bioinformatics Institute (BII), ASTAR, Singapore, Singapore; 11 Human Potential Translational Research Programme, National University of Singapore, Singapore, Singapore; 12 Institute of Global Governance and Innovation for a Shared Future, City University of Hong Kong, Hong Kong, China (Hong Kong)

**Keywords:** COVID-19, social media surveillance, Twitter, natural language processing, real-time analysis, sentiment analysis, virus mutations, geographic tracking

## Abstract

**Background:**

The global outbreak of COVID-19 has significantly impacted health care systems and has necessitated timely access to information for effective decision-making by health care authorities. Conventional methods for collecting patient data and analyzing virus mutations are resource-intensive. In the current era of rapid internet development, information on COVID-19 infections could be collected by a novel approach that leverages social media, particularly Twitter (subsequently rebranded X).

**Objective:**

The aim of this study was to analyze the trending patterns of tweets containing information about various COVID-19 symptoms, explore their synchronization and correlation with conventional monitoring data, and provide insights into the evolution of the virus. We categorized tweet sentiments to understand the predictive power of negative emotions of different symptoms in anticipating the emergence of new Omicron subvariants and offering real-time assistance to affected individuals.

**Methods:**

Relevant user tweets from 2022 containing information about COVID-19 symptoms were extracted from Twitter. Our fine-tuned RoBERTa model for sentiment analysis, achieving 99.7% accuracy for sentiment analysis, was used to categorize tweets as negative, positive, or neutral. Joinpoint regression analysis was used to examine the trends in weekly negative tweets related to COVID-19 symptoms, aligning these trends with the transition periods of SARS-CoV-2 Omicron subvariants from 2022. Real-time Twitter users with negative sentiments were geographically plotted. A total of 105,934 tweets related to fever, 120,257 to cough, 55,790 to headache, 101,220 to sore throat, 3410 to vomiting, and 5913 to diarrhea were collected.

**Results:**

The most prominent topics of discussion were fever, sore throat, and headache. The weekly average daily tweets exhibited different fluctuation patterns in different stages of subvariants. Specifically, fever-related negative tweets were more sensitive to Omicron subvariant evolution, while discussions of other symptoms declined and stabilized following the emergence of the BA.2 variant. Negative discussions about fever rose to nearly 40% at the beginning of 2022 and showed 2 distinct peaks during the absolute dominance of BA.2 and BA.5, respectively. Headache and throat-related negative sentiment exhibited the highest levels among the analyzed symptoms. Tweets containing geographic information accounted for 1.5% (1351/391,508) of all collected data, with negative sentiment users making up 0.35% (5873/391,508) of all related tweets.

**Conclusions:**

This study underscores the potential of using social media, particularly tweet trends, for real-time analysis of COVID-19 infections and has demonstrated correlations with major symptoms. The degree of negative emotions expressed in tweets is valuable in predicting the emergence of new Omicron subvariants of COVID-19 and facilitating the provision of timely assistance to affected individuals.

## Introduction

Symptom surveillance is crucial for public health and monitoring the transmission dynamics of infectious diseases [1]. Surveillance involves indicator-based and event-based surveillance [2], of which the former uses established protocols to collect structured data based on correlations between cases and hospital diagnostic data. Currently, the identification and variation of virus symptoms are studied in large populations in hospitals. However, delays in testing and reporting among institutions impact the timeliness of decision-making, hinder the prompt mobilization of resources and public education after determining the symptoms of mutated viruses, and potentially lead to losses and public panic [3].

Event-based surveillance can be performed using nonconventional data sources, such as social media, news, and public health networks, and excels in the timely identification of emerging public health issues. There is a trend of growing reliance on social media and search engines for pandemic-related data collection. Health systems are integrating conventional and advanced techniques to obtain more immediate and comprehensive data, which enhances the formulation of health policy.

Since the H1N1 influenza epidemic in 2009, social media has started to play a pivotal role in surveillance. For instance, HealthMap combines formal sources such as the World Health Organization (WHO) and the Centers for Disease Control and Prevention (CDC) with informal data sources such as Twitter to reduce reporting delays [4]. A study on the 2010 cholera outbreak in Haiti assessed the correlation between unofficial data sources (eg, HealthMap and Twitter) and government-reported cases. The effective reproduction number was also estimated using these nonofficial sources [5]. Twitter has also been applied to monitor dengue fever in analyses focusing on various aspects of tweets to identify potential cases [6], especially sociodemographic characteristics [7].

Social media surveillance has continued to progress for other infectious diseases, including Ebola [8], Middle East respiratory syndrome [9], and Zika [10]. Retrospective prediction models based on Google and Twitter data have demonstrated potential in the early detection of suspected Zika cases [10]. In the era of the COVID-19 pandemic, research has shifted focus toward understanding risk factors for the disease [11], its symptoms and mental health impacts [12], and using sentiment analysis to combat the spread of misinformation [13]. Mackey et al [14] used machine learning to identify COVID-19 symptoms and related experiences from Twitter, highlighting testing challenges and underreporting issues. A recurrent neural network has also been used to classify sentiment and analyze manifestations [15].

Wu et al [16] demonstrated the dynamic changes of COVID-19 symptoms over time, from the early typical respiratory symptoms to the later musculoskeletal and nervous system symptoms. We propose a Twitter-based symptom surveillance method for COVID-19 infections focusing on 6 typical symptoms. We chose Twitter (subsequently rebranded as X) as a data source to collect public information on people with COVID-19 from 2022. A previous study collected a large number of tweets related to the Omicron variant and classified them based on sentiment, language, source, and type of tweet, among other aspects [17]. We also analyzed the sentiment of their tweets to determine the severity of the impact for each individual. Our model can detect the emotions of users with COVID-19 on a geographic map in real time. This method monitors the dynamic epidemiological situation, especially the evolution of symptoms of Omicron subvariants, and explores correlations with public sentiment. The findings could assist in making timely governmental decisions for strengthening pandemic prevention and control.

## Methods

### Overview

This study used quantitative and qualitative methods for collecting and analyzing tweets related to COVID-19 to synchronize, forecast, and track the evolution of the different COVID-19 symptoms. The focus was on symptoms, the emotions of those who have been infected, and geographical information. We identified tweets related to COVID-19 symptoms as our key search criterion and collected tweets containing any reference to the occurrence of the disease and its symptoms from January 1, 2022, to December 31, 2022. The data were prepared in 3 steps: (1) data selection, (2) data collection, and (3) data cleaning.

### Data Selection

We collected tweets related to COVID-19 symptoms using the following key search terms: “fever,” “cough,” “headache,” “throat,” and “vomit,” which are related to common symptoms of COVID-19 according to the CDC in October 2022 [18]. We collected data if any of these terms and the term “COVID” appeared in a published tweet. We chose daily Twitter data from 2022 because of its higher maturity in test technologies and monitoring systems, which supported the meaningful comparison between Twitter data and clinical data.

### Data Collection

During the data collection phase, daily tweets in English were gathered from Twitter using Python scripts. The advanced search function on Twitter was used to extract comprehensive datasets, followed by a data-validation process to maintain collection accuracy. Additional data crawling was conducted to reinforce the integrity of the dataset. Official numbers of weekly confirmed cases were reported by the WHO [19]. We used 3 continuous modules to analyze COVID-19 symptoms for the real-time surveillance of people infected with COVID-19: temporal analysis, sentiment analysis, and user-feature analysis.

### Sentiment Analysis

After data collection, we preprocessed the data of each tweet using Python (Python Software Foundation) and R (The R Foundation), which filtered out tweets with significant amounts of garbled characters before inputting them into the sentiment analysis model. The sentiments expressed in short tweets are generally more direct, which makes it easier to determine the sentiment category. To accurately determine their sentiment, both training and inference were conducted on all tweets using the latest natural language processing (NLP) model, the RoBERTa base model [20], in the Hugging Face repository.

We identified the tweets of users who were infected with COVID-19, predicted the probability of different types of emotions in each tweet (positive, neutral, and negative), and selected the category with the highest probability as the sentiment result. We used Omicron subvariants data from the weekly epidemiology reports of the WHO to create a geographical map of Omicron subvariants’ proportions over 1 year. The WHO data are obtained through geographical coverage and align with our worldwide Twitter data. The accuracy of sentiment analysis for the 3 categories was 99.7%.

We performed a joinpoint regression to analyze changes in counts of weekly negative tweets. The subvariant transition periods are set as the week during which a new Omicron subvariant replaces the previous subvariant, plus 1 week before and after. We aim to identify whether the significant joinpoints captured by the joinpoint regression fall within these subvariant transition periods.

### Geographical Analysis

In the completed map, each red dot represents a tweet related to COVID-19 symptoms. Geographic information about Twitter users is obtained by using the Twitter API to see what geographic information exists, or by analyzing metadata or tweets to infer geographic information. The map provides a quick display of the daily count of negative tweets and the tally of tweets concerning COVID-19 in a specific region while summarizing the negative, positive, and neutral sentiments. A line graph shows the fluctuations in the quantity of COVID-19–related tweets regionally over time.

### Ethical Considerations

This study did not involve human participants or interventions, and thus no informed consent was required. The analysis relied solely on publicly available Twitter data, ensuring the anonymity of individuals. Any personal information collected was handled in accordance with ethical guidelines, and no identifiable user data are disclosed in this paper.

## Results

### Real-Time Analysis of Tweets Related to COVID-19 Symptoms From 2022

**Figure 1.**
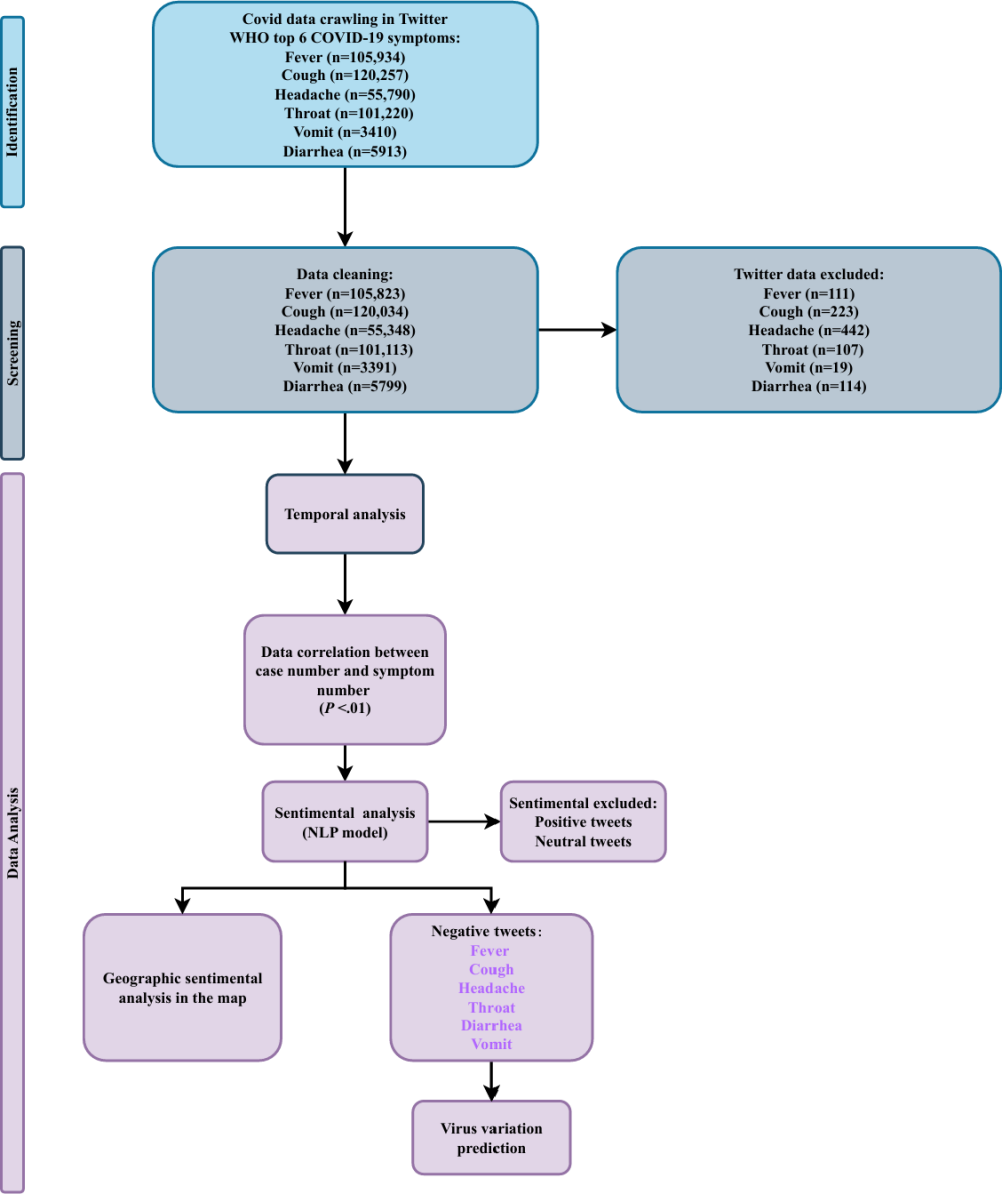
A flowchart of screening for COVID-19 symptom–related tweets. NLP: natural language processing; WHO: World Health Organization.

The collected data encompassed 6 symptoms mentioned in tweets, of which 105,934 tweets were related to fever, 120,257 were related to cough, 55,790 were related to headache, 101,220 were related to sore throat, 3410 were related to vomiting, and 5913 were related to diarrhea (Figure 1).

**Figure 2.**
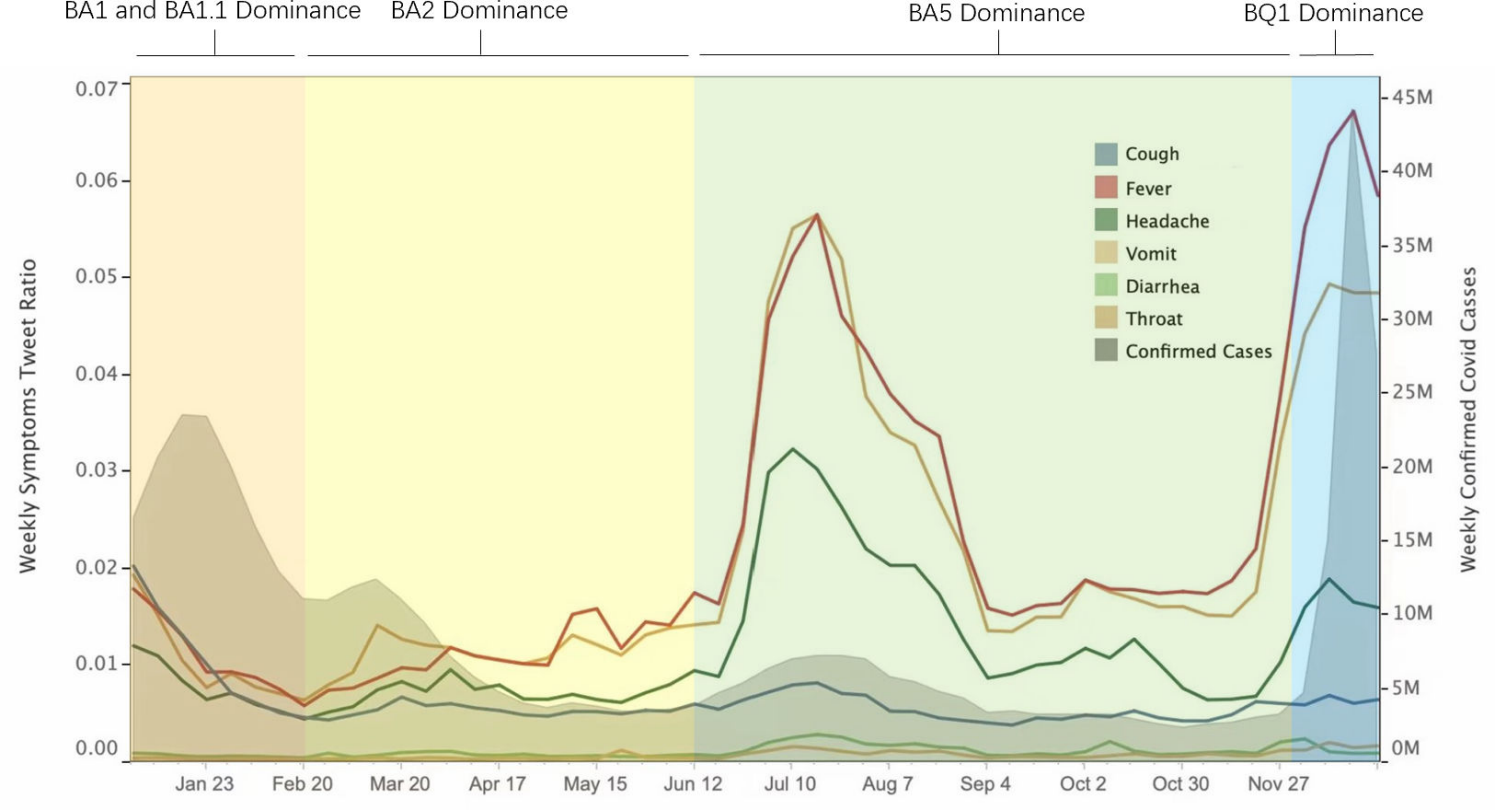
Weekly average severity of daily tweets about symptoms and confirmed cases.

Figure 2 shows the trend of the weekly ratio of symptom-related tweets and new cases of COVID-19 worldwide. We divided the year 2022 into 4 periods based on the prevalence of different variants. There were 4 peaks in the number of new cases throughout the year, which occurred in January, March, July, and December. Furthermore, the fluctuations in ratios of weekly symptom tweets demonstrated a certain level of synchronous pattern with the evolution of Omicron variants and waves of new cases.

According to the gathered symptom surveillance data, discussions surrounding fever, sore throat, and headache were the most prominent, which was consistent with real-world observations. Fever was common from the beginning of the pandemic, whereas sore throat and headache became more prevalent during the Omicron period [21,22] and attracted increased attention from multiple public health authorities [23].

In Figure 2, the periods of analysis are as follows: (1) period 1 (January 1-February 20): dominance of BA.1 and BA.1.1, (2) period 2 (February 20-June 12): BA.2 dominance, (3) period 3 (June 12-December 1): BA.5 dominance, and (4) period 4 (December 1-12): BQ1 and XBB dominance. The weekly average of daily symptom severity is the calculated number of tweets for each symptom as a proportion of tweets for all symptoms. Gray shaded area: weekly confirmed cases from the WHO. To match the 2 values of the vertical axis (weekly average daily), we performed vertical axis matching to display the image.

The confirmed cases (Figure 2, gray shaded area) increased only in the first 2 weeks during period 1 and then continued to decrease because this period was in the later stages of the dominance of the BA.1 and BA.1.1 subvariants. Restrictions on travel and activities and vaccination measures were implemented as early as November-December 2021. Correspondingly, the tweets related to all symptoms demonstrated a decreasing trend with little difference among them.

During period 2, the BA.2 subvariant emerged as the dominant strain and superseded BA.1 and BA.1.1. Although the increased transmissibility of BA.2 [24] contributed to a second peak in confirmed cases, the risk of reinfection with BA.2 was lower [24], and the severity did not escalate compared to earlier periods. Consequently, there were some slight fluctuations in Twitter discussions. At the same time, tweets related to sore throat, fever, and headache began to dominate during this period.

By mid-2022, in period 3, the symptom surveillance data showed a peak that corresponds to a surge in new cases in the real world, particularly in terms of fever, throat discomfort, and headache. Tweets related to other symptoms also exhibited relatively elevated peaks compared to their baseline levels. During this period, the BA.5 subvariant swiftly supplanted BA.2 as the predominant strain. Notably, BA.5 was characterized by enhanced immunity and heightened transmissibility [25-28].

Some research indicates that individuals infected with the BA.5 subvariant are more prone to experiencing several common symptoms that we have selected [29], which reinforces the robustness of our analysis. Heightened public sensitivity on social media outpaced the actual occurrence of the disease and may have been influenced by sore throats becoming a primary symptom and growing awareness of the Omicron subvariants. The dynamic interplay between subvariant characteristics and public discourse is evident.

In period 4, the BQ1 subvariant dominated, which was accompanied by the emergence of XBB. Research indicates that BQ.1 and XBB have higher effective reproduction numbers [30] and transmissibility compared to BA.2 and BA.5 [31]. A peak of newly reported cases in December 2022 occurred in the week of December 5-11 and reached its highest point in the week of December 19-25. The Twitter data promptly revealed this conspicuous peak, and the top 3 most discussed symptoms were more sensitive.

### Sentiment Analysis of Tweets Related to COVID-19 Symptoms From 2022

Among the 8 subvariants, BA.1, BA.1.1, BA.2, BA.5, and BQ.1 notably dominated at various points during 2022. At the beginning of the year, BA.1 had already started to be gradually replaced by BA.1.1, but BA.2 quickly emerged and maintained a proportion greater than 50% for an extended period of 16 weeks. Subsequently, BA.5 began to replace BA.2 as the dominant subvariant, sustaining a proportion greater than 50% for an even longer duration of 20 weeks. By the end of the year, BQ.1 surpassed BA.5, while XBB also emerged.

Negative discussions about fever rose to nearly 40% at the beginning of the year and subsequently exhibited 2 distinct peaks during the periods when BA.2 and BA.5 were absolutely dominant, respectively. The prompt public response to the symptom of fever during the early stages of subvariant evolution may be attributed to its role as the first symptom to manifest after infection with COVID-19 [32].

**Figure 3.**
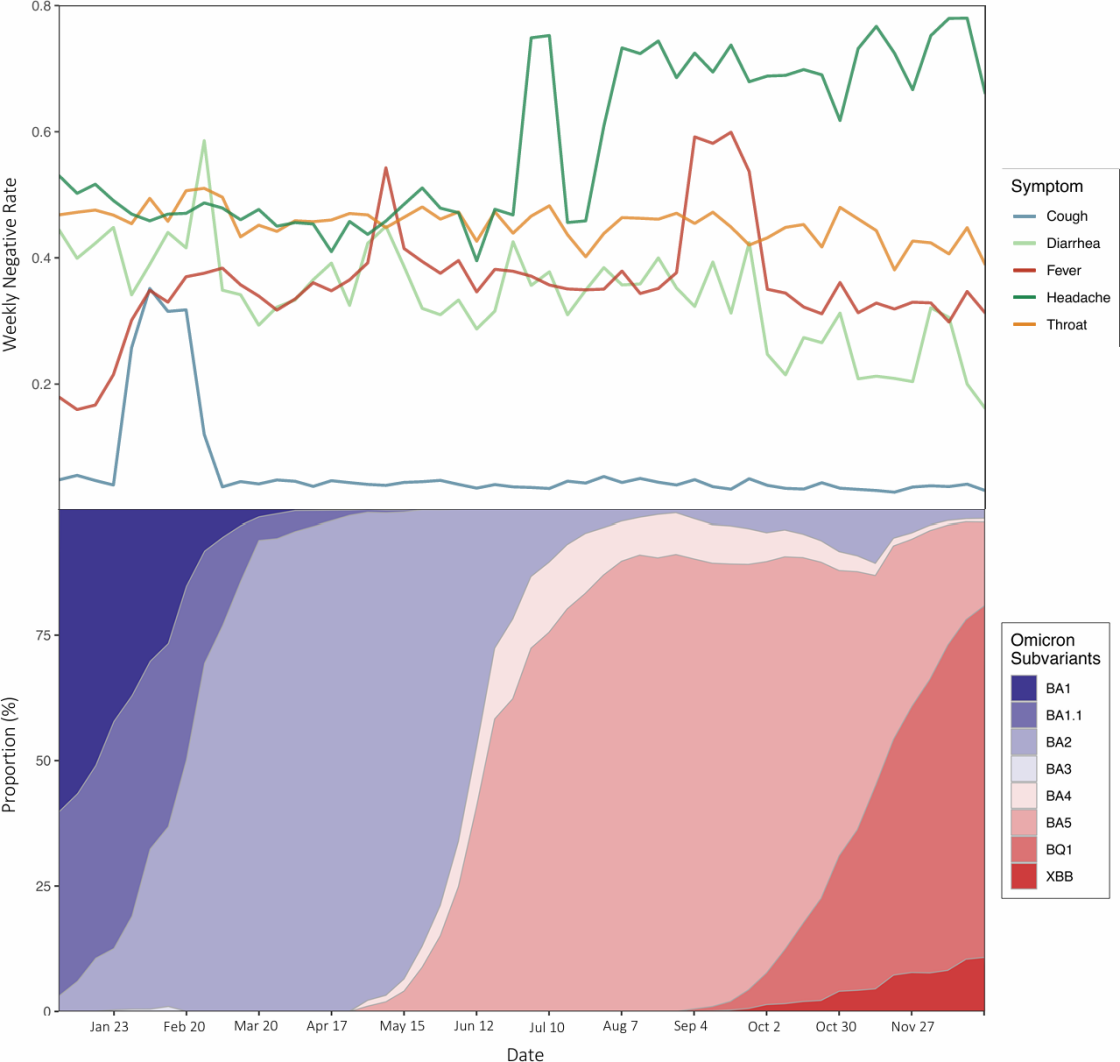
Specific symptom sentiment analysis with proportions of Omicron subvariants. The weekly negative rate is the calculated number of negative tweets for each symptom as a proportion of all tweets for the specific symptom. The evolution of the proportions of Omicron subvariants was determined based on the data on Omicron subvariant proportions from World Health Organization weekly reports.

One characteristic of the Omicron subvariants is that the majority of symptoms closely resemble those of the cold, including headaches and sore throat [33]. This is illustrated in Figure 3, where these 2 symptoms exhibit the highest levels of negative sentiment.

### Joinpoint Regression of the Number of Negative Tweets of COVID-19 Symptoms From 2022

**Figure 4.**
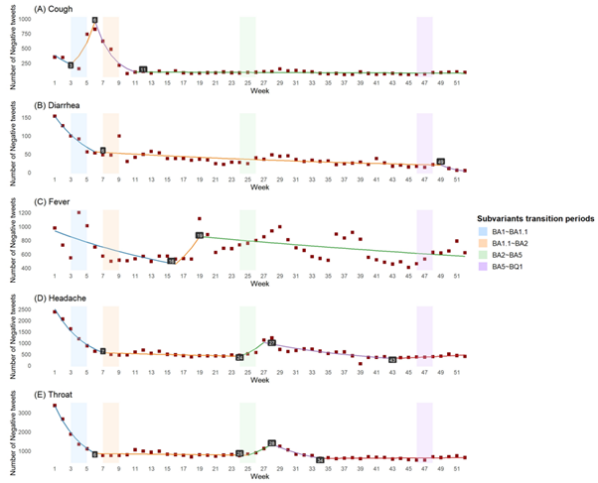
Joinpoint regression analysis of weekly negative tweets: alignment with subvariant transition periods. Significant joinpoints, which indicate a significant change in the trend of negative tweet counts, are highlighted with the specific week in black squares. The background’s shaded regions represent the transition periods of major SARS-CoV-2 Omicron subvariants throughout 2022.

Cough exhibited a transient rise in negative sentiment during only the initial stages of BA.2, while negative attitudes in this regard remained consistently low and stable during other periods (Figure 4). Given its persistent presence as a symptom of COVID-19 throughout the pandemic, the public might have developed a relatively comprehensive understanding of both the symptom itself and its associated management strategies. This symptom typically manifests in the middle stages of the infection [32], and its prevalence had already begun to decline with the Omicron variant [33].

The joinpoint analysis revealed similar trends for diarrhea, headache, and throat-related discussions (Figure 4). These discussions declined as the proportion of BA.1 decreased and stabilized before BA.2 became dominant. However, only headache and throat-related negative discussions exhibited minimal fluctuations during the later phase of BA.2-BA.5.

Fever was able to capture more of the subvariants’ evolution, with negative tweets exhibiting a peak in sentiment during the later stages of each subvariant transition (Figure 4). Model estimates of the joinpoint regression can be seen in Multimedia Appendix 1.

### An Interactive Map of Opinion Analysis and Sentiment Visualization of COVID-19 Tweets

**Figure 5.**
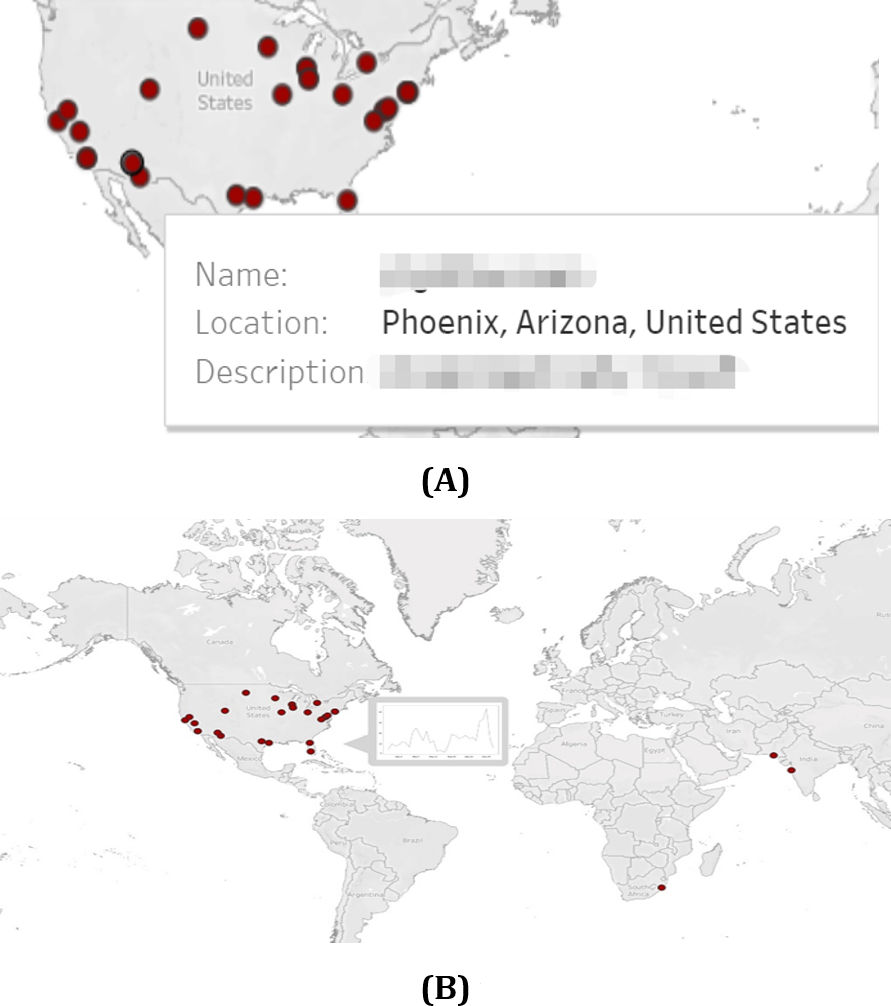
(A) An interactive map of opinion analysis and sentiment visualization of COVID-19 tweets showing a sample of users with negative tweets. (B) Users can click on a specific location on the map to obtain detailed information about emotions from comments about COVID-19.

Social media platforms have become an essential channel for people to express their views and emotions about the epidemic. We developed an interactive map demo using the United States as an example to collect Twitter data, perform sentiment analysis through NLP, and link the results to corresponding geographic locations (Figure 5A). Of all the data we collected, tweets containing geographic information accounted for 1.5% (1351/391,508), while negative sentiment users accounted for 0.35% (5873/391,508) of all related tweets. This demo link is available at Tableau Public [34]. This system provides a visual representation that provides an intuitive understanding of attitudes and emotions toward COVID-19 across different regions in the United States. Users can click on a specific location on the map to obtain detailed information about emotions from comments about COVID-19 (Figure 5B).

The interactive map could deepen the understanding of people’s opinions and emotions and provide valuable national information for decision-making, opinion management, and social intervention. In decision-making and public opinion management, analyzing the distribution of different opinions and emotional tendencies in the United States could help decision-makers better understand attitudes and opinions about COVID-19 in different places. This could help to formulate more effective policies to meet the needs of the public and improve the acceptance of policies. In terms of social intervention, interactive maps of the United States could help the government better understand the distribution of views. As a result, state governments could implement more targeted interventions, such as providing psychological support, disseminating information, and mobilizing communities to address mental health and social issues related to COVID-19.

## Discussion

### Principal Findings

Our findings corroborate clinical observations that Omicron subvariants shifted symptom profiles toward cold-like manifestations (eg, sore throat and headache), consistent with reports by Menni et al [21] and Schulze et al [22]. The heightened negative sentiment around headache and sore throat (Figure 3) likely reflects their unexpected prevalence during Omicron, contrasting with earlier variants. Notably, fever-related negativity proved uniquely sensitive to subvariant evolution, exhibiting dual peaks during BA.2 and BA.5 dominance. This suggests public social media reactivity may serve as a leading indicator for viral evolution—a novel extension of prior infodemiology studies [eg, 16,17]. However, the sustained high negative sentiment toward symptoms such as headache (Figure 3) requires nuanced interpretation. While headache prevalence increased during Omicron acute infection [21], its persistent discussion throughout 2022 may also reflect growing public awareness of Long COVID, where headache is a predominant symptom affecting most cases [35,36]. This “twin-demic” context, where acute infection waves coexist with Long COVID burden, introduces complexity in attributing symptom trends solely to variant dynamics.

The real-time geographic dashboard (Figure 5) operationalizes a key advantage of social media surveillance: overcoming delays in conventional reporting. For instance, our system detected the December 2022 case surge 1‐2 weeks before WHO confirmation [19], enabling proactive resource targeting. This addresses critical gaps identified in traditional systems, such as lags in resource allocation during BA.5’s rapid spread [31]. Furthermore, sentiment-driven resource allocation (eg, directing mental health support to regions with rising negative tweets) offers a scalable complement to clinical surveys. Our method also expands on Mackey et al’s [14] symptom detection framework by integrating sentiment as a proxy for symptom severity and subvariant impact, though future models should distinguish acute versus chronic symptom manifestations through keyword filtering (eg, “long-hauler” and “post-COVID”) or duration-based classification.

Our study has 4 key limitations. First, the exclusive reliance on 2022 English-language tweets introduces demographic and linguistic biases, as Twitter users skew toward younger, urban, technologically adept populations. This limits generalizability to non-English speakers, older adults, and rural communities. Future work must integrate multilingual platforms (eg, Weibo and Facebook) and extend temporal coverage to strengthen model robustness. Second, clinical generalizability is constrained by the underrepresentation of pediatric populations. While children exhibit distinct Omicron symptom severity [10], our adult-dominated dataset cannot capture this nuance. Subpopulation-specific analyses require complementary clinical data.

Third, geographic sparsity remains a challenge: only 1.5% (1351/391,508) of tweets contained usable location data, and inferred geodata may introduce biases. Resource allocation based solely on these sparse signals risks misprioritization. Cross-validation with electronic health records and refined geolocation inference algorithms is essential. Fourth, global representativity is compromised by overlapping biases in Twitter data (overrepresenting Anglophone nations) and WHO variant surveillance (skewed toward high-income countries). The observed correlations between sentiment and subvariants may reflect region-specific epidemiological patterns rather than global dynamics. Future research must prioritize intentional sampling from underrepresented regions and multiplatform data fusion to validate cross-cultural applicability. In addition, our analysis did not explicitly account for Long COVID–related symptom reporting. Given that 2022 marked a peak in Long COVID cases (WHO COVID-19 Weekly Epidemiological Update, July 2022), symptom discussions, particularly for neurological manifestations like headache, likely conflated acute infection and chronic sequelae. This conflation may partially explain the sustained high negative sentiment for certain symptoms beyond variant transition periods (eg, headache negativity remained >30% during BA.5 decline in Figure 3).

Beyond COVID-19, this study establishes a scalable framework for infodemiology-driven pandemic response. The integration of real-time social media analytics with conventional surveillance creates a synergistic system capable of (1) accelerating outbreak detection (eg, leveraging symptom sentiment as an early-warning signal), (2) optimizing precision public health interventions (eg, dynamic resource allocation based on geotagged emotional hotspots), and (3) enabling rapid evaluation of policy impacts (eg, monitoring sentiment shifts postvaccine rollout). As novel pathogens emerge, such systems could mitigate the “infodemic” paradox, where data abundance coexists with decision-making delays, by converting public discourse into actionable intelligence. Future iterations will expand linguistic parameters, incorporate clinical correlates, and develop predictive algorithms to establish a global public health intelligence platform, ultimately bridging the gap between digital epidemiology and frontline response. The framework’s adaptability is particularly valuable for diseases with long-term sequelae (eg, Long COVID), where real-time tracking of chronic symptoms through social discourse could accelerate therapeutic development and patient support.

### Conclusions

In summary, our method allows rapid surveillance of changes in the geographical COVID-19 symptoms. This was accomplished using sentiment analysis of tweets from people who have been infected with COVID-19 to detect the geographical severity rate. At the individual level, it can detect the real-time severity of COVID-19 and could make it possible to provide medical and mental assistance. It also provides a new solution for coping with the urgent emergence of a new pandemic.

## Supplementary material

10.2196/66237 Multimedia Appendix 1 Model estimates of joinpoint regression.

## Abbreviations

CDC: Centers for Disease Control and Prevention
NLP: natural language processing
WHO: World Health Organization
