# Supplementary material for: Surveillance of Twitter Data on COVID-19 Symptoms During the Omicron Variant Period: A Sentiment Analysis
Source: JMIR Form Res. 2025 Sep 23;9:e66237. doi: 10.2196/66237 (PMC12456870; doi:10.2196/66237)
Supplement: Multimedia Appendix 1 [file formative-v9-e66237-s001.pdf]

Supplementary table 1. Model estimates of joinpoint regression

Annual Percent Change (APC)							
**Symptoms**	**Lower Endpoint**	**Upper Endpoint**	**APC**	**Lower CI**	**Upper CI**	**Test Statistic (t)**	***p*-value**
Cough	1	3	-21.9325	-45.6704	12.1772	-1.3794	0.175257
Cough	3	6	60.6999	4.9027	146.1754	2.2462	0.030143
Cough	6	11	-36.6381	-45.2789	-26.6329	-6.2855	< 0.000001
Cough	11	52	-0.5685	-1.2485	0.1162	-1.6778	0.101
**Average Annual Percent Change (AAPC)**							
Cough	1	52	-3.0646	-6.0915	0.06	-1.9229	0.054491
**Annual Percent Change (APC)**							
**Symptoms**	**Lower Endpoint**	**Upper Endpoint**	**APC**	**Lower CI**	**Upper CI**	**Test Statistic (t)**	***p*-value**
Diarrhea	1	6	-18.1612	-26.5518	-8.812	-3.734	0.000538
Diarrhea	6	49	-2.3287	-3.0565	-1.5955	-6.3492	< 0.000001
Diarrhea	49	52	-41.2609	-75.6678	41.7988	-1.2167	0.230194
**Average Annual Percent Change (AAPC)**							
Diarrhea	1	52	-6.8364	-11.5416	-1.881	-2.6781	0.007404
**Annual Percent Change (APC)**							
**Symptoms**	**Lower Endpoint**	**Upper Endpoint**	**APC**	**Lower CI**	**Upper CI**	**Test Statistic (t)**	***p*-value**
Fever	1	16	-4.6809	-7.2128	-2.08	-3.5889	0.00083
Fever	16	19	23.2345	-39.9585	152.9371	0.5856	0.561163
Fever	19	52	-1.2278	-2.0402	-0.4086	-3.0145	0.004261

**Average Annual Percent Change (AAPC)**							
Fever	1	52	-0.9756	-5.0647	3.2896	-0.4557	0.648625
**Annual Percent Change (APC)**							
**Symptoms**	**Lower Endpoint**	**Upper Endpoint**	**APC**	**Lower CI**	**Upper CI**	**Test Statistic (t)**	***p*-value**
Headache	1	7	-21.9621	-26.3131	-17.3542	-8.7503	< 0.000001
Headache	7	24	-1.864	-3.9187	0.2346	-1.8002	0.079775
Headache	24	27	35.5997	-18.671	126.0851	1.206	0.23528
Headache	27	43	-7.1062	-9.0973	-5.0714	-6.887	< 0.000001
Headache	43	52	4.4289	-1.1874	10.3646	1.587	0.12081
**Average Annual Percent Change (AAPC)**							
Headache	1	52	-3.2471	-6.36	-0.0307	-1.9784	0.047887
**Annual Percent Change (APC)**							
**Symptoms**	**Lower Endpoint**	**Upper Endpoint**	**APC**	**Lower CI**	**Upper CI**	**Test Statistic (t)**	***p*-value**
Throat	1	6	-23.9476	-27.2232	-20.5246	-12.5877	< 0.000001
Throat	6	25	-0.5539	-1.5233	0.4251	-1.1478	0.258213
Throat	25	28	20.544	-7.953	57.8634	1.4024	0.168915
Throat	28	34	-12.5855	-17.8319	-7.0041	-4.3994	0.000085
Throat	34	52	0.3113	-0.7661	1.4003	0.5826	0.563574
**Average Annual Percent Change (AAPC)**							
Throat	1	52	-3.2117	-4.952	-1.4395	-3.5263	0.000421
